# Exploring the Influence of Built Environment on Car Ownership and Use with a Spatial Multilevel Model: A Case Study of Changchun, China

**DOI:** 10.3390/ijerph15091868

**Published:** 2018-08-29

**Authors:** Xiaoquan Wang, Chunfu Shao, Chaoying Yin, Chengxiang Zhuge

**Affiliations:** 1 MOE Key Laboratory for Urban Transportation Complex Systems Theory and Technology, Beijing Jiaotong University, Beijing 100044, China; 15120886@bjtu.edu.cn; 2 Key Laboratory of Transport Industry of Big Data Application Technologies for Comprehensive Transport, Beijing Jiaotong University, Beijing 100044, China; 3 Department of Geography, University of Cambridge, Downing Place, Cambridge CB2 3EN, UK; zgcx615@126.com

**Keywords:** car ownership, car use, built environment, spatial autocorrelation, multilevel Bayesian model

## Abstract

Although the impacts of built environment on car ownership and use have been extensively studied, limited evidence has been offered for the role of spatial effects in influencing the interaction between built environment and travel behavior. Ignoring the spatial effects may lead to misunderstanding the role of the built environment and providing inconsistent transportation policies. In response to this, we try to employ a two-step modeling approach to investigate the impacts of built environment on car ownership and use by combining multilevel Bayesian model and conditional autocorrelation (CAR) model to control for spatial autocorrelation. In the two-step model, the predicting car ownership status in the first-step model is used as a mediating variable in the second-step car use model. Taking Changchun as a case study, this paper identifies the presence of spatial effects in influencing the effects of built environment on car ownership and use. Meanwhile, the direct and cascading effects of built environment on car ownership and use are revealed. The results show that the spatial autocorrelation exists in influencing the interaction between built environment and car dependency. The results suggest that it is necessary for urban planners to pay attention to the spatial effects and make targeted policy according to local land use characteristics.

## 1. Introduction

Car dependency is one of the most influential contributing factors to air pollution, traffic congestion, and energy consumption [1]. Additionally, it is widely believed that car use can increase the risk of health problem due to more sedentary behavior than other travel modes [2]. A growing body of literature has focused on the link between built environment and travel behavior in order to reduce car dependency through promoting sustainable urban planning strategies in developed countries [3,4,5]. It is also viewed as a long-term effective solution to the negative effect of car dependency on the environment to promote high-density and compact urban development strategies due to the likelihood to engage in active travel. Especially in developing country like China, many cities are experiencing urban sprawl with urbanization process, thus increasing more motorized travel demand and transport-related environmental issues. Reducing car ownership and use has recently become the emerging national concern [6]. On the other hand, it is a good opportunity for policy makers and urban planners to shape the interaction between the built environment and car dependency in developing countries due to the changes in built environment along with rapid urbanization [7].

However, to the best of our knowledge, few studies have been conducted in developing countries although many studies have investigated the link between built environment and travel behavior [8,9]. China is the largest developing country and experiencing an explosive increase in motorized travel demand with rapid urbanization in recent years. These dramatic transformations lead to a different situation where empirical studies in Western cities could provide few evidences [10]. Second, a very limited number of empirical studies have paid attention to the influence of spatial effects on the link between built environment and car dependency [11,12,13]. Although some existing studies have paid increasing attention to spatial context and attempted to capture the spatial heterogeneity by applying multilevel models, it is still a challenge to address spatial autocorrelation, which is important for capturing the potential correlation of observations located in nearby locations in the geographic data context. The study contributes to the literature by addressing the spatial effects when investigating the influence of built environment on car ownership and use in Changchun, China. To achieve this, we employed a two-step model based on Changchun Household Travel Survey, in which the multilevel Bayesian model combined with conditional autocorrelation (CAR) model is used to address spatial autocorrelation.

The remainder of the paper is organized as follows. In Section 2, we review related studies on the effects of built environment and the spatial effects. Then, we describe the data in Section 3, while Section 4 presents the methodology used for this study. Section 5 presents the model results, followed by policy implications and future work in Section 6.

## 2. Literature Review

### 2.1. Built Environment, Other Factors, and Car Dependency

Over the past few decades, important conclusions have been reached on the interaction between the built environment and travel behavior [14,15,16,17,18]. In the existing studies, it is acknowledged that the built environment mainly consists of physical and social elements that make up the structure of a community and it can influence travel behavior. Additionally, the built environment has been summarized as “D variables”, developing from “three Ds” defined by Cervero [19] to “six Ds” in recent studies including diversity, density, design, destination accessibility, distance to transit, and demand management [20,21]. Numerous existing studies have also confirmed that the built environment plays a remarkable role in car ownership and use decision [1,3,4,22,23,24,25,26]. Although some debatable conclusions are reached in existing studies, some built environment characteristics show significant influences, directly or indirectly, on a range of outcomes including car ownership, mode choice, vehicle miles traveled (VMT), vehicle hours traveled (VHT), car trip frequencies et al. Especially in the context of Chinese cities, the influence of built environment on car dependency is attracting more and more attention due to the concerns about environmental and health issues brought by the rapid growth of car ownership and use. For instance, Jiang et al. [26] employed the multinomial logistic model and double-hurdle model to investigate the effect of the land use and street characteristics on car ownership and use in Jinan respectively and found that along with the proximity to regional transport infrastructures, most land use characteristics could influence the car dependency. Additionally, Li et al. [1] found that higher land use mix and accessibility of living facilities could help reduce the car dependency for people living near metro stations in Beijing.

Apart from built environment, a body of other factors has been found to be significantly associated with car ownership and use, including individual factors, household factors, travel-related factors and self-selection factors. Moreover, some of these factors may be more influential than the built environment factors. Many previous studies have explored the influence of socio-economic characteristics on car ownership and use, in which household income is found to be one of the most key factors [27,28,29]. The influence of household structure is also well studied from different aspects, consisting of household size [30], household workers [31], household children [25], and household composition [32]. Hukou is a special system conducted in China to ensure the reasonable migration, which is a term that attracts the attention of scholars due to its relation with urbanization [33]. Hukou is a population policy to control the movement of the rural population into the city. It is also a direct factor on the distribution of the state’s welfare. In some Chinese cities, it can determine purchase qualification of house and car.

Additionally, travel-related factors have a significantly association with car dependency, which is also confirmed by some existing studies [7,18,34]. Moreover, the self-selection effect, which is characterized by the phenomenon that residents would choose their residence location according to their preferences for travel mode and land use patterns, can influence car dependency further [35,36]. For example, Hong et al. examined the relationship between built environment and travel behavior, in which self-selection was found to have significant influence on household VMT [37]. Additionally, Cao et al. [38] used Guangzhou as a case and found that built environment and self-selection effects influenced car ownership and commuting distance, further producing influence on emissions.

### 2.2. Spatial Effects

Spatial autocorrelation is an explanation for the phenomenon that observations at nearby locations tend to have similar characteristics, which is documented in the literature [37,39,40,41]. The observations are not independent when spatial autocorrelation occurs. Therefore, statistical methods ignoring spatial effects may lead to inefficient or even biased estimated results due to their assumption that the observations are independent. For instance, Bhat [11] employed a multilevel cross-classified model to analyze commuting model choice considering spatial clustering of observations. The results indicate that spatial clustering exists and should be taken into account to avoid inferior data fit. To address the challenge and incorporate the spatial context, researchers have developed several methods to resolve the problem of spatial autocorrelation in spatial data [42,43,44]. For instance, Wu et al. [13] conducted two rounds of surveys to identify the effect of public transit improvement on car dependency and found that spatial dependency existed between adjacent neighborhoods. In another example, Wang et al. [40] employed a Poisson log-normal conditional autoregressive model to investigate the determinants of safety impacts of roadway network and found that spatial autocorrelation existed in crashes on highways. In addition, the spatial autocorrelation has also been studied in land use and emission analysis. For instance, Hong et al. [41] examined the relationship between residential density and transportation emissions and utilized a multilevel Bayesian model with spatial random effects to address the spatial autocorrelation. The results suggest that spatial autocorrelation can influence the effect of residential density on emission. Eboli et al. employed the geographically weighted regression model to evaluate transit service quality considering spatial variation of passengers’ responses across the study area and it could provide more appropriate results compared with ordinary least square model [45]. Additionally, Eboli et al. found the existing clusters of similar values in the distribution of the service quality attributes based on passenger satisfaction survey data from Milan [46].

However, there are few efforts having been made to handle the spatial autocorrelation existing in influencing the role of the built environment to play in car ownership and use decision. In response to this, this study will contribute to current literature in two aspects. First, statistic models have their own disadvantages in capturing spatial effects, which may lead to a biased estimation. To address the spatial autocorrelation, we employ a two-step Bayesian multilevel model with spatial random term to explore the determinants of car ownership and use in this study. Second, there are many differences between China and Western countries, including the level of economic development, urbanization, hukou system, and the culture context. Although there is a growing body of literature investigating the influence of built environment on car dependency in developed countries, studies about the impacts in China are away from reaching the consensus. What’s more, rapid urbanization in Chinese cities provides a good chance to explore the link between built environment and car dependency.

Based on our literature review, we identified a number of research gaps which are detailed below: (1) from the influential variable side, previous studies are mainly conducted in developed countries and not much attention has been paid to the influential variables about China-specific issues like hukou [47]; (2) From the methodology side, previous studies use discrete choice model [6,17,31,48], structural equation model (SEM) [4,25], and regression model [1,26,49,50] to investigate the relationship between land use and transport characteristics. SEM can take into the mediating effects of car ownership when investigating the influencing factors, but have a limitation of recognizing the spatial effects. Additionally, although discrete choice methods and regressions methods can model the spatial effects, they cannot take into account mediating effects. In this study, a two-step modeling approach with spatial random effects is proposed to investigate the determinants of car ownership and use and address the spatial effects in the unified analytical framework.

## 3. Data and Variable

### 3.1. Study Region

**Figure 1:**
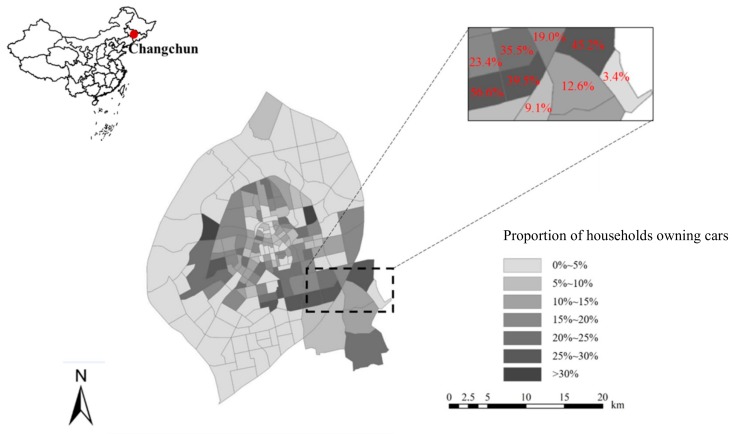
Study region and traffic analysis zones.

The study region in this study is Changchun city as shown in Figure 1, which is a mid-sized city in Northeast China. It covers approximately 20,565 km^2^ and has more than 7 million people [51]. As the capital of Jilin province, Changchun continues to exhibit economic growth and urban sprawl which are relative to the rapid urbanization process in China. Additionally, motorized travel demand has explosively grown over the past decade due to the rapid economic growth and urban expansion in Changchun. Changchun has been chosen as a member of “Transit Metropolis” program and invested huge amounts of capital in public transit construction in order to reduce car dependency. However, similar to many Chinese cities, the growth in car ownership and use still leads to notorious traffic congestion and air pollution. Therefore, Changchun is chosen as the study region to provide references for similar cities in China.

### 3.2. Data and Descriptive Statistics

The primary data used for the empirical explosion is extracted from the 2012 Changchun household travel survey conducted by Beijing Transport Institute. The survey is part of a comprehensive traffic model report undertaken by Beijing Transport Institute and Changchun Institute of Urban Planning and Design to monitor comprehensive traffic network, travel demand, and travel behavior. The survey is conducted from 1 May 2012 to 13 May 2012. The survey provided socio-economic characteristics consisting of household income, household size, hukou type. Additionally, completed travel information of all members in the respondent’s household was collected on the assigned day, including travel modes, trip purposes, departure time, arrival time, and origin and destination of a trip. In the dataset, travel information of 20,000 households is available. About 18.2% of the total sample owned one or more cars. As shown in Figure 1, the proportion of households that own at least one car in the traffic analysis zones is presented. After error-checking and clearing the raw data, a total of 100,058 complete trip records of 16,732 households are used in this study. Socio-economic characteristics are described in Table 1.

Built environment measurements are collected from two major sources: AMAP.com and Changchun traffic map. As shown in Figure 1, there are 237 traffic analysis zones (TAZs) in the study region and the average area of each TAZ is 2.46 km^2^. The data reflects five dimensions of built environment characteristics, including population density, intersection density, transit station density, distance to central business district (CBD), and land use mix at the TAZ level. Intersection density is obtained based on Changchun traffic map using the ArcGIS platform (Environmental Systems Research Institute, Redlands, CA, USA) and only four-way intersections are used in this analysis. Transit station density is measured by the ratio of the number of bus stops and metro stations within the TAZ. Distance to CBD represents the location of residence, which is measured based on the Euclidean distance between the household’s TAZ centroid and CBD. Due to the limitations of data acquisition, the entropy index was used based on the point of interest (POI) to measure land use mix, following Cao et al. [38]. The POIs were extracted from AMAP includes residential buildings, hotels, restaurants, supermarkets, parks, squares, malls, schools, hospitals, banks, and government departments. The index is a measurement of the distribution evenness of different land use types in a given TAZ.

(1) $$\mathrm{Land}\;\mathrm{use}\;\mathrm{mix}=-\sum_{i=1\;}^{N}p_{i}\ln p_{i}/\ln N$$

where *i* corresponds to POI types and *p_i_* is the proportion of a specific POI type from the total area of a given TAZ. *N* is the total number of possible POI types. The index value ranges from 0 to 1 and a higher value means a more balanced land use pattern in the TAZ.

The descriptive statistics of built environment measurements are described in Table 2.

## 4. Methodology

The analysis was twofold. First, we used household characteristics and built environment characteristics to predict the household car ownership status using a Bayesian multilevel discrete choice model. In the first-step model, car ownership was used as a binary variable and treated as the dependent variable. Then we analyzed the determinants of household VMT, in which the predicted car ownership derived from the first model was used as a mediating variable instead of the observed car ownership status. The built environment and socio-economic characteristics were treated as independent variables. The two-step model can address the potential endogeneity bias and selection bias [52,53]. The endogeneity bias results from the endogeneity between car ownership and use due to the influence of unobserved factors on both car ownership and use. The second bias can be due to the fact that car use only happens when the household owns cars. Additionally, the two-step modeling approach can explicitly distinguish the direct effects of exogenous variables and indirect effects via car ownership on car use simultaneously [26,31]. Moreover, the proposed models assume that observations at nearby locations tend to have similar characteristics and TAZs vary as a function of built environment variables measured at the TAZ level [30]. The CAR model is used to specify the spatial autocorrelation. The detail models are described as follows.

In the first-step model, we performed a Bayesian multilevel discrete choice model on household car ownership by incorporating CAR model to address the spatial effects. In the model, we treated the household car ownership status as a binary variable and used relevant socio-economic and built environment characteristics as the independent variables. The socio-economic and built environment characteristics are treated as household and TAZ level variables respectively because households living in the same TAZ share a common environment. The car ownership model takes the Bayesian multilevel discrete choice model form, and the utility function is as below.

(2) $$\begin{array}{l} U_{ih\;}=\alpha_{|i|h}+\beta_{SD}^{T}X_{ih}^{SD}+s_{|i|h}+\varepsilon_{ih}\\ \alpha_{h}=\varphi +\gamma_{BE}^{T}X_{h}^{BE}+\sigma_{h}\\ s_{h}=N(\bar{s_{h}},\frac{\sigma_{s}^{2}}{n_{h}})\\ \bar{s_{h}}=\sum_{k\in \mathrm{neighborhood}}\bar{w_{h,k}}s_{k}/n_{h} \end{array}$$

where $U_{ih}$ is the utility function of household *i* residing in TAZ *j* owning one or more cars. $X_{ih}^{SD}$ and $X_{h}^{BE}$ are the socio-economic and built environment characteristics, respectively. $\alpha_{|i|h}$ is the varying intercept. $\beta_{SD}^{T}$ and $\gamma_{BE}^{T}$ are the vectors of parameters to be calibrated. The spatial autocorrelation term is represented by $s_{|i|h}$, which means residents at nearby locations behave similarly due to their similar unobserved characteristics. $s_{h}$ is assumed to follow a normal distribution in this study. $\varepsilon_{ih}$ is the error term and assumed to follow a Gumbel distribution. $\bar{w_{h,k}}s_{k}$ is an element in the spatial adjacent matrix, representing the adjacent relation between TAZ *h* and *k*. $n_{h}$ is the number of TAZs sharing common boundaries with TAZ *h*.

In this study, the household car ownership is treated as a binary variable according to whether the household owns cars or not. The household car ownership decision can be described as follows.

(3) $$y_{ih\;}=\left\{ \begin{array}{l} 1,\;\mathrm{if}\;U_{ih}>U_{jh},\forall j\in A\\ 0,\;\mathrm{otherwise} \end{array}\right.$$

where $y_{ih}$ is the choice indicator. If the household owns cars, $y_{ih}$ takes the value of one, and zero otherwise.

Then, the probability of household *i* owning one or more cars can be obtained as follows.

(4) $$p_{ih\;}(y_{ih}=1|X_{ni}^{SE},X_{ni}^{BE},X_{ni}^{PA},CAR_{ni},TD_{ni},s_{h},\varepsilon_{ni},\sigma_{h})=\frac{\exp(\varphi +\gamma_{BE}^{T}X_{h}^{BE}+\beta_{SD}^{T}X_{ih}^{SD}+\sigma_{h}+s_{|i|h})}{\sum \exp(\varphi +\gamma_{BE}^{T}X_{h}^{BE}+\beta_{SD}^{T}X_{ih}^{SD}+\sigma_{h}+s_{|i|h})}$$

In this study, common boundary matrix is used to measure the adjacent relation between two TAZs as below.

(5) $$w_{h,k\;}=\left\{ \begin{array}{l} \mathrm{the}\;\mathrm{length}\;\mathrm{of}\;\mathrm{common}\;\mathrm{boundary},\;\mathrm{if}\;\mathrm{TAZ}\;h\;\mathrm{is}\;\mathrm{adjacent}\;\mathrm{to}\;\mathrm{TAZ}\;k\\ 0,\mathrm{otherwise} \end{array}\right.$$

The spatial adjacent matrix can be obtained through standardizing the elements in common boundary matrix according to min-max normalization scheme.

Therefore, the predicted car ownership status can be calibrated as below.

(6) $$X_{ih\;}^{CAR}=\left\{ \begin{array}{l} 1,\;\mathrm{if}\;p_{ih}>1-p_{ih}\\ 0,\;\mathrm{otherwise} \end{array}\right.$$

where $X_{ih}^{CAR}$ is the predicted car ownership status of household *i* living in TAZ *h*.

In the second-step model, a normal regression model with spatial random effects was employed to explore the determinants of car use. In the model, the dependent variable was derived based on all car-based trips conducted by the household members on the assigned day. According to the origin and destination of the trip in the dataset, vehicle kilometers traveled (VKT) was calibrated based on the shortest path on the road network. It is worth mentioning that log VKT was chosen as the dependent variable in this study because the VKT was found to be positively skewed to the right. In addition, the predicted car ownership status was used as an exogenous variable in the car use model. The model can address the potential influences of the exogenous variables, including the socio-economic and built environment characteristics, thus the indirect influence of socio-economic and built environment characteristics via car ownership can be revealed simultaneously. Therefore, the final car use model is as follows.

(7) $$\begin{array}{l} y_{i}\sim N(\omega_{|i|h\;}+\beta_{SD}^{T}X_{ih}^{SD}+\beta_{CAR}^{T}X_{ih}^{CAR}+v_{|i|h},\;\sigma_{y}^{2})\\ \omega_{h}\sim N(\varphi +\gamma_{BE}^{T}X_{h}^{BE},\sigma_{h}^{2})\\ v_{h}=N(\bar{v_{h}},\frac{\sigma_{v}^{2}}{n_{h}})\\ \bar{v_{h}}=\sum_{k\in \mathrm{neighborhood}}\bar{w_{h,k}}v_{k}/n_{h} \end{array}$$

where $X_{ih}^{CAR}$ is the predicted car ownership status for household *i* living in in TAZ *h*.

To estimate the car ownership model and car use model, multilevel Bayesian procedure based on the Markov Chain Monte Carlo (MCMC) method was conducted, which could overcome the deficiency resulting from the maximum likelihood estimation method [42,54]. The estimation method is based on Bayes’ Theorem as follows.

(8) $$\pi (\theta |y)=\frac{L(y|\theta )\pi (\theta )\;}{\int L(y|\theta )\pi (\theta )d\theta }$$

where *y* is a vector of observed variables. θ is the parameter vector of likelihood function. π(θ|y) is the posterior distribution under given *y*. L(y|θ) is the likelihood function. ∫L(y|θ)π(θ)dθ is the edge probability distribution of the observed variables. π(θ) is the prior distribution. Based on the posterior distribution of parameters, MCMC method can generate a chain to make point and interval estimations through successive sampling [54,55]. Different from p value estimation based on the mean and the variance, MCMC method provides a more direct way through the posterior distributions of parameters. Moreover, the uncertainty can be obtained based on the MCMC method because it can provide a specific CI (Confidence Interval) for the estimated parameters. In this analysis, the mean of estimated parameters and 95% CI is presented instead of p value. It presents the 95% CI by providing the lower bound of 2.5% and upper bound of 97.5%. If the 95% CI does not include zero, it means that the influence of the corresponding independent variable on dependent variable is significant.

## 5. Result and Discussion

### 5.1. Car Ownership Model

The estimation result for car ownership model is presented in Table 3. With regard to spatial autocorrelation term, the parameter $\sigma_{s}$ is found to be significant at the 95% significance level, which demonstrates that spatial autocorrelation exists in car ownership decision. The result confirms that households living in nearby areas tend to have similar decision on purchasing cars, which indicates that unobserved autocorrelation could moderate the influence of built environment on households’ car ownership behavior.

According to Table 3, most coefficients of socio-economic characteristics show significant influences on household car ownership. For instance, it is found that the influence of hukou on car ownership is significantly positive, indicating that households with local hukou have a higher probability of owning cars. This may be explained by that hukou system ensure that households with local hukou enjoy better social welfares in China. Additionally, the results reveal that higher household income increases the likelihood of owning cars, which is consistent with existing studies [1,56,57]. Because many Chinese families still cannot afford to purchase a car [26], household income still serves as one of the primary determinants of household car ownership. Household size is found to have no significant influence on household car ownership. Although existing studies suggest that household size can increase the likelihood of owning cars [31], bigger household size means an increasing net income, thus decreasing the travel budget. Finally, household student has a positive influence on household car ownership at the significance level of 95%, indicating that the number of household students increases the likelihood of owning cars. This may be explained by that most parents tend to show concerns about the safety of children on the way to school and would like to drive them to school in China.

Turning to the built environment characteristics, residential density shows a significantly negative influence on car ownership. This means that households living in lower residential density areas are more likely to own cars because communities with higher residential density generally mean better living facilities in neighborhoods, thus reducing the motorized travel demand. Land use mix is found to be negatively associated with car ownership. It means that, when a household lives in a TAZ with compact land use, the probability of owning cars decreases. Distance to CBD shows no significant influence on car ownership, which is different from the existing study [34]. However, the existing studies also produce mixed results about the influence [31,34,57]. On one hand, living farther from CBD means a longer commuting distance due to the fact that most employments concentrate around CBD. Therefore, people living in these areas have to choose motorized mode because of the longer distance between origin and destination, thus increasing the likelihood of owning cars. On the other hand, commuting is only a factor that could influence car ownership. The other factors, including economical factor, should also be considered. Transit station density is found to have a significantly negative influence on car ownership. Also, higher intersection density reduces the likelihood of owning cars because higher intersection density generally provides a friendlier environment for active travel mode.

### 5.2. Car Use Model

The estimation result of car use model is presented in Table 4. The influence of $\sigma_{v}$ at the 95% significance level suggests that spatial autocorrelation exists. The result indicates that households living in nearby areas have similar car use behavior.

The predicted household car ownership status, which is derived from the first-step model according to Equation (6) and estimated parameters, shows a strongly positive influence on the household total VKT. The result suggests household car ownership can affect household car use. Also, it is also found that hukou has significantly positive influence on household VKT. It is possible for the reason that households with local hukou generally tend to enjoy better welfares and have higher requirement for travel convenience and effectiveness. Similar with previous studies, the result shows that household income is positively associated with household car use. Compared with the influence of household size on car ownership, the result suggests that although household size shows no significant influence on car ownership, the households with bigger household size generate more car use. This may be explained by that bigger household size could generate more motorized travel demand such as education and social interaction purpose, but meanwhile bigger household size means more other household expenses and a limited budget for travel, thus constraining car-purchasing decision. Finally, household student on car use is not related with household VKT at the significance level of 95%, which is different from that in car ownership model. This indicates that the number of household students merely affects car use via the influence on car ownership.

After controlling for socio-economic factors, several built environment characteristics also show significant influence on car use. For instance, living in areas with higher residential density tends to be associated with a lower likelihood of car use. This may be due to the fact that areas with higher residential density generally have better living facilities to meet the demand of daily life and thus reduce the travel demand further. Similar with household student, land use mix and intersection density are both not associated with car use at the 95% significant level, yet they are associated with car ownership at the 95% significant level. In addition, distance to CBD has a positive influence on household car use at the 95% significant level, which suggests that multi-center compact development strategy may be an effective way to reduce VKT. Finally, it is found that as living in areas with higher transit station density is significantly associated with a lower likelihood of car use. This result suggests that investing on public transit may help reduce car use.

### 5.3. Combined Effects of Built Environment

To investigate the combined effects of built environment on household car use, including direct and indirect effects via car ownership, we estimated the cascading influences via simulation. Measuring elasticities of household car use with respect to the built environment characteristics are presented in Table 5, which are calibrated by combining the two models (the detailed calculation method can be seen in notes of Table 5).

The estimated elasticities of VKT show that distance to CBD is the most influential contributing factor to VKT with a combined elasticity of 0.12. This means that households living farther from CBD tend to generate more VKT and the influence is much greater than the other built environment characteristics. Therefore, it is necessary to promote tailor-made urban planning strategies according to the locations. Additionally, compared with the rest characteristics, transit station density could present relatively larger elasticities, indicating that transit station density is an important determinant of car use. Specifically, if we double the transit station density, it would reduce 8 percent of household VKT. Additionally, other built environment characteristics all may play a remarkable role in influencing car use. Therefore, the results suggest that promoting sustainable land use strategies can reduce car use effectively in urban areas.

## 6. Conclusions

This study aims to examine the direct and cascading effects of the built environment on car ownership and use based on a two-step model, in which CAR model is combined to the Bayesian multilevel model to address the spatial effects. Based on data from Changchun, the study can provide insightful results for the literature from two aspects: methodology implementations and policy implications.

First, a two-step model can provide an insight into the link between the built environment and travel behavior by revealing the direct and cascading effects of built environment on car ownership and use. Meanwhile, Bayesian multilevel model combined with CAR model provides evidence for the role of spatial effects play in influencing the impacts of built environment on car dependency. The results suggest that it is important to accommodate spatial autocorrelation that can moderate the influence of built environment on individual decision-making.

Second, as for policy implications, this paper provides concrete evidence on the interaction between built environment and car ownership and use for urban planners. The results indicate that promoting dense land use and transit-oriented development can reduce car ownership and use. In addition, communities nearer from CBD and with higher accessibility decline the car dependency for residents. However, it should be noted that one-size-fits-all design should not be the solution to reduce car dependency due to the fact that car ownership and use can vary over space. Therefore, urban planners should acknowledge the spatial effects and find the most suitable built environment sets according to the local land use characteristics.

The study also has some limitations. First, due to data limitation, self-selection effect is not addressed in the study. We control for the socio-demographics and spatial effects, but more attitude data is needed in future to address the self-selection effects. Second, it would be helpful to compare empirical results for spatial effects across cities because the spatial effects may vary with different cities.

## Figures and Tables

**Table 1:** Descriptive statistics of socio-economic and travel-related characteristics.

Variable Name	Variable Description	Min	Max	Mean
Car ownership	1, if one or more cars are available; 0, otherwise	0	1	0.18
Hukou	1, local hukou; 0, otherwise	0	1	0.95
Household income 1	1, household income yearly is less than 20,000 (RMB); 0, otherwise (around US$3 thousand)	0	1	0.25
Household income 2	1, household income yearly is between 20,000–100,000 (RMB); 0, otherwise (around US$3–15 thousand)	0	1	0.73
Household income 3	1, household income yearly is less than 100,000 (RMB); 0, otherwise (around US$15 thousand)	0	1	0.02
Household size	Number of household members	1	9	2.71
Household student	Number of household students	0	4	0.33

**Table 2:** Descriptive statistics of built environment characteristics.

Variable Name	Variable Description	Mean	Standard Deviation
Population density	Population density per square kilometer at the TAZ level	0.34	0.22
Intersection density	Intersection density per square kilometer at the TAZ level	0.59	0.17
Transit station density	Transit station density per square kilometer at the TAZ level	10.50	5.91
Distance to CBD	Euclidean distance from residence to CBD (unit: km)	4.8	2.91
Land use mix	A measure of the composition of residential buildings, hotels, restaurants, supermarkets, parks, squares, malls, schools, hospitals, banks, and government departments	33.38	17.83

Note: CBD: central business district.

**Table 3:** Multilevel Bayesian Logistic regression of household car ownership.

Variable	Mean	95% CI	
		2.5%	97.5%
Socio-demographics at household level			
Hukou	0.91	0.79	1.04
Household income 1 (reference: Household income 2)	−0.17	−0.25	−0.09
Household income 3 (reference: Household income 2)	0.43	0.30	0.56
Household size	0.03	−0.05	0.11
Household student	0.08	0.04	0.12
Built environment at TAZ level			
Residential density	−0.51	−0.31	−0.71
Land use mix	−0.23	−0.37	−0.10
Distance to CBD	0.09	−0.03	0.23
Transit station density	−0.09	−0.14	−0.04
Intersection density	−0.08	−0.14	−0.02
$\sigma_{h}$	0.09	0.07	0.11
$\sigma_{s}$	1.23	0.76	1.71

Note: CI: confidence interval. TAZ: traffic analysis zone.

**Table 4:** Multilevel Bayesian Normal regression of household VKT (vehicle kilometers traveled).

Variable	Mean	95% CI	
		2.5%	97.5%
Socio-demographics at household level			
Predicted car ownership status	2.19	1.85	2.53
Hukou	0.07	0.04	0.11
Household income 1 (reference: Household income 2)	−0.19	−0.28	−0.11
Household income 3 (reference: Household income 2)	0.39	0.18	0.61
Household size	0.05	0.01	0.12
Household student	0.42	−0.09	0.93
Built environment at TAZ level			
Residential density	−0.10	−0.17	−0.03
Land use mix	−0.07	−0.15	0.01
Distance to CBD	0.05	0.01	0.09
Transit station density	−0.12	−0.17	−0.08
Intersection density	−0.11	−0.32	0.10
$\sigma_{h}$	0.29	0.09	0.49
$\sigma_{v}$	0.18	0.12	0.23

**Table 5:** Elasticities of household VKT with built environment variables.

Variable	Elasticity of VKT via Car Ownership	Combined Elasticity of VKT
Residential density	−0.01	−0.02
Land use mix	−0.01	−0.01
Distance to CBD	−	0.12
Transit station density	−0.03	−0.08
Intersection density	−0.04	−0.04

Note: Adapted from [31], we used the $VKT_{baseline}$ and $VKT_{new}$ represent the baseline total VKT generated and new VKT estimated after applying 10% increase for the variable of interest. The $VKT_{baseline}$ is obtained using the coefficient estimates from the regression model (Table 4), in which the predicted car ownership status is used. Then a 10% increase of the target variable and we update the new status of the predicted car ownership status according to the discrete choice model. $VKT_{new}$ is generated by using the regression coefficients and the predicted number of car ownership. The elasticity can be calibrated by $\left((VKT_{new}-VKT_{baseline})/VKT_{baseline})/(0.10\right)$.
